# Tristetraprolin Down-Regulation Contributes to Persistent TNF-Alpha Expression Induced by Cigarette Smoke Extract through a Post-Transcriptional Mechanism

**DOI:** 10.1371/journal.pone.0167451

**Published:** 2016-12-02

**Authors:** Xue-Ke Zhao, Pulin Che, Ming-Liang Cheng, Quan Zhang, Mao Mu, Hong Li, Yuan Luo, Yue-Dong Liang, Xin-Hua Luo, Chang-Qing Gao, Patricia L. Jackson, J. Michael Wells, Yong Zhou, Meng Hu, Guoqiang Cai, Victor J. Thannickal, Chad Steele, J. Edwin Blalock, Xiaosi Han, Ching-Yi Chen, Qiang Ding

**Affiliations:** 1 Department of Infectious Diseases, The Hospital Affiliated to Guizhou Medical University, Guiyang, Guizhou, China; 2 Department of Medicine, Division of Pulmonary, Allergy and Critical Care Medicine, University of Alabama at Birmingham, Birmingham, Alabama, United States of America; 3 Department of Oral Surgery, Shanghai Stomatology Hospital, Fudan University, Shanghai, China; 4 Department of Infectious Diseases, Public Health Center of Guiyang, Guiyang, Guizhou, China; 5 Department of Infectious Diseases, People's Hospital of Guizhou Province, Guiyang, Guizhou, China; 6 Neurology, University of Alabama at Birmingham, Birmingham, Alabama, United States of America; 7 Department of Biochemistry, University of Alabama at Birmingham, Birmingham, Alabama, United States of America; USDA-ARS, UNITED STATES

## Abstract

**Rationale:**

Tumor necrosis factor-alpha (TNF-α) is a potent pro-inflammatory mediator and its expression is up-regulated in chronic obstructive pulmonary disease (COPD). Tristetraprolin (TTP) is implicated in regulation of TNF-α expression; however, whether TTP is involved in cigarette smoke-induced TNF-α expression has not been determined.

**Methods:**

TTP expression was examined by western blot analysis in murine alveolar macrophages and alveolar epithelial cells challenged without or with cigarette smoke extract (CSE). TNF-α mRNA stability, and the decay of TNF-α mRNA, were determined by real-time quantitative RT-PCR. TNF-α protein levels were examined at the same time in these cells. To identify the molecular mechanism involved, a construct expressing the human beta-globin reporter mRNA containing the TNF-α 3’-untranslated region was generated to characterize the TTP targeted site within TNF-α mRNA.

**Results:**

CSE induced TTP down-regulation in alveolar macrophages and alveolar epithelial cells. Reduced TTP expression resulted in significantly increased TNF-α mRNA stability. Importantly, increased TNF-α mRNA stability due to impaired TTP function resulted in significantly increased TNF-α levels in these cells. Forced TTP expression abrogated the increased TNF-α mRNA stability and expression induced by CSE. By using the globin reporter construct containing TNF-α mRNA 3’-untranslated region, the data indicate that TTP directly targets the adenine- and uridine-rich region (ARE) of TNF-α mRNA and negatively regulates TNF-α expression at the post-transcriptional level.

**Conclusion:**

The data demonstrate that cigarette smoke exposure reduces TTP expression and impairs TTP function, resulting in significantly increased TNF-α mRNA stability and excessive TNF-α expression in alveolar macrophages and epithelial cells. The data suggest that TTP is a novel post-transcriptional regulator and limits excessive TNF-α expression and inflammatory response induced by cigarette smoke.

## Introduction

Cigarette smoke exposure has been firmly associated with the development of chronic obstructive pulmonary disease (COPD) [1–4]. COPD remains a major public health problem in the world and is one major leading cause of chronic morbidity and mortality in the United States [1–4]. COPD is predicted to become the third leading cause of death by 2020 in the United States and globally according to world health organization. COPD is a progressive lung disease and patients have reduced lung function due to the abnormal permanent enlargement of airspaces and destruction of lung structure [1;4–6]. The pathological features of COPD include, but not limited to, substantial inflammation, fibrotic remodeling of the airways, and alveolar destruction [2;3–6].

Persistent inflammation is thought as the main driving force of COPD pathogenesis [4;7–10]. Increased expression of pro-inflammation mediators contributes to persistent inflammation associated with inflammation cell recruitment, epithelial cell death, and abnormal enlargement of airspace in COPD [7;8;11]. Additionally, evidence shows that inflammatory responses continue despite cessation of smoking in COPD patients [3;12–14]. However, molecular mechanisms leading to the persistent inflammation in COPD lungs have not been completely defined.

Tumor necrosis factor alpha (TNF-α) is one of the potent pro-inflammation mediators and its expression is up-regulated in COPD lungs [15–17]. Increased TNF-α expression has been tightly linked to persistent inflammatory responses in COPD lungs, and evidence supports that TNF-α plays an important role in cigarette smoke induced inflammation in COPD [8;15–16]. Regulation of mRNA stability is one of the important mechanisms to control gene expression and plays a role in inflammatory response to tissue injury [18–19]. However, the molecular mechanism of regulating TNF-α expression at the mRNA level in response to cigarette smoke exposure is not well understood.

Tristetraprolin (TTP) is a widely distributed phospho-protein encoded by the immediate–early response gene [20–23]. TTP was initially identified and characterized as a transcription factor, because TTP was found to regulate gene expression through modulation of the level of mRNA [20–28]. In fact, the function of TTP is to destabilize mRNA as an mRNA-destabilizing protein rather than regulating gene expression through transcription [23–28]. Published studies implicated that TTP regulates TNF-α expression [19;21;27]. It has been demonstrated that TTP binds to the 3’-untranslated region (UTR) of TNF-α mRNA and regulates TNF-α expression by decreasing TNF-α mRNA levels [19;21;27]. The phenotype of TTP-deficient mice further supports the role of TTP in mediating TNF-α expression, as well as the potential role of TTP as an anti-inflammatory protein *in vivo*. Mice deficient in TTP developed a severe syndrome characterized by growth retardation and cachexia, arthritis, dermatitis, and autoimmunity [27]. Treatment of TTP-deficient mice with neutralizing anti-TNF-α antibodies resulted in alleviating most of the pathological features in TTP-deficient mice [27], demonstrating the involvement of TNF-α in the development of the pathological features in TTP-deficient mice. These published data strongly suggest that impaired TTP function could lead to persistent and excessive TNF-α expression. However, the effects of cigarette smoke on TTP expression and on TTP’s ability to limit TNF-α expression have not been well studied.

This study aims to investigate whether cigarette smoke extract exposure dampens TTP function and whether impaired TTP function contributes to the increased TNF-α expression induced by cigarette smoke extract exposure. The results show that cigarette smoke extract exposure reduced TTP expression in alveolar macrophage and alveolar epithelial cells. Reduced TTP expression leads to significantly increased stability of TNF-α mRNA, and results in increased and prolonged expression of TNF-α in alveolar macrophage and alveolar epithelial cells. Exogenous TTP overexpression abrogated the increased stability of TNF-α mRNA in macrophages and epithelial cells exposed to cigarette smoke, resulting in significantly reduced TNF-α protein levels in these cells. Furthermore, the data revealed the molecular mechanism involved. TTP binds to and directly targets the adenine- and uridine-rich region (ARE) of TNF-α mRNA and negatively regulates TNF-α expression at the post-transcriptional level. Taken together, the data demonstrate that cigarette smoke exposure impairs TTP function resulting in prolonged TNF-α mRNA stability and persistent TNF-α expression. TTP is a novel post-transcriptional regulator and limits the TNF-α expression and pro-inflammatory response induced by cigarette smoke.

## Materials and Methods

### Reagents

Research cigarettes (3R4F) were purchased from the center for tobacco reference products at the University of Kentucky (Lexington, KY). The following purified antibodies were purchased: anti-TTP, anti- human influenza hemagglutinin (HA) tag, anti-glyceraldehyde 3-phosphate dehydrogenase (GAPDH), and anti- green fluorescent protein (GFP) (Santa Cruz Biotechnologies, Santa Cruz, CA, USA), biotinylated rat anti-mouse anti-CD-45 (R&D systems, Minneapolis, MN), biotinylated rat anti-mouse anti-CD-16/32 (BD Biosciences Pharmingen, San Jose, CA). Enzyme-linked immunosorbent assay (ELISA) kits to determine the TNF-α protein level were purchased from the R&D System (Minneapolis, MN). All other reagents were purchased from Sigma (St. Louis, MO, USA) or Fisher Scientific (Waltham, MA, USA).

### Cigarette smoke extract (CSE)

CSE was used to study the effects of *in vitro* cigarette smoke exposure as described previously [29–30]. All CSE solutions were freshly prepared. Research cigarettes were directly connected to one end of the test tube and the other end of the tube is emerged in DMEM/RPMI medium in one vacuum glass vessel. The cigarettes were smoked and the mainstream smoke was passed through 30 ml medium (pre-warmed to 37°C) by application of a vacuum to the vessel containing the DMEM medium. Each cigarette was smoked for about 5 min. Three cigarettes were used to generate 30 ml of CSE solution as described [29]. The CSE was diluted with DMEM medium. Final concentrations are expressed as % volume/volume. CSE concentrations 4% and 8% were chosen in the present study based on our optimization data (data not shown) that they were sufficient to induced significant TTP downregulation. Based on previous report [29], these approximately correspond to exposures associated with smoking slightly less than 1 pack per day to slightly more than 1.5 packs per day of cigarettes. The CSE control medium (for CSE’s effect) was prepared with the same protocol above except that the cigarettes were not lit and were not burned.

### Cell and cell culture

Primary alveolar macrophages were isolated from C57Bl/6 mice (Jackson Laboratory) and performed as described [31–32]. The research has been approved by local Animal Care and Use Committee (IACUC). Briefly, after mice were sacrificed, a 20-gauge catheter was inserted into the trachea and the lungs were lavaged six times with 1 ml of ice-cold saline. The recovered lavage fluids were centrifuged at 200 x g at 4°C for 3 min. The supernatants were decanted, and the numbers of cells in the pellets counted. The cells were then washed once with RPMI 1640 with 10% fetal bovine serum (FBS) and were allowed to adhere for 2 hours in RPMI with 10% FBS. After 2 hours, the nonadherent cells were removed along with the supernatant. Greater than 90% of the lavaged and harvested cells were excluded from trypan blue and > 95% of the cells were determined to be alveolar macrophages by morphologic appearance. The murine alveolar macrophage cell line (MH-S) was purchased from the American Type Culture Collection (ATCC, Manassas, VA) and cultured as per manufacturer’s instruction. Primary type II alveolar epithelial cells were derived from mice and cultured by the procedures described previously [33]. The rat alveolar epithelial cells (L2) were purchased from ATCC and were propagated and maintained in F-12K medium supplemented with 10% FBS and 100 units/ml penicillin/streptomycin as per ATCC instructions and as described [34].

### Adenoviral vectors

Generation, amplification, and utilization of the replication incompetent adenoviral vectors were described previously [35–36]. Briefly, the replication-deficient adenoviral vectors containing TTP or GFP cDNA were generated by using the Adeno-X expression system 2 according to the manufacturer’s instructions (Clontech, Mountain View, CA, USA). The adenoviral vectors were rescued and amplified in 293 cells, and purified by CsCl gradient centrifugation.

### Plasmids

The construct expressing the human beta (β)-globin reporter mRNA containing the TNF-α ARE (pTRE-hGB-TNF), the pTRE empty control construct, and the pTet-Off construct were described previously by us [37]. Briefly, to express human β-globin reporter mRNAs containing the adenine- and uridine-rich region (ARE) of TNF-α mRNA, a polylinker (5’-AGATCTATCGATCTGCAGGATATCGCGGCCGCGTCGACAAGCTTGCATGC-3’) was inserted into a BglII site immediately downstream of the stop codon of the β-globin gene, which was previously subcloned into a tetracycline (Tet)-regulatory vector, pTRE (BD Biosciences). The TNF-α ARE (nucleotides 1221 to 1310; GenBank accession no. M10988) was then inserted between EcoRV and HindIII sites and infected cells as described previously by us [37;38].

### mRNA stability, mRNA half-life, and real time quantitative RT-PCR

mRNA levels were determined by real-time quantitative RT-PCR and mRNA stability was evaluated through measurement of half-life as described by us previously [39]. Half-life of mRNA was determined by examining the amount of remaining mRNA at each time point as indicated. Briefly, cells were treated with actinomycin D (10 μg/ml) to block new transcription as described by us previously [39]. Immediately after addition of actinomycin D, cells were treated with CSE or controls for the indicated time and harvested. Total RNA was collected, and the amounts of TNF-α or G3PDH mRNA at each indicated time points were quantified by real-time RT-PCR as described [38]. Samples were assayed in triplicate and the values were normalized to the relative amounts of G3PDH mRNA levels within the same samples at the same indicated time points.

### ELISA assays to determine protein expression of TNF-α

Enzyme-linked immunosorbent assay (ELISA) kits were used to determine the TNF-α protein levels per manufacture’s instructions.

### Western Blotting

Western blot assays were performed as described by us previously [36]. Briefly, equivalent micrograms of whole cell lysates were electrophoresed on a disulfide-reduced 7.5% SDS-PAGE, transferred to Immobilon-P membrane (Millipore Corp., Bedford, MA, USA), probed with indicated antibodies and developed with ECL system (Pharmacia Biotech, Piscataway, NJ, USA). The expression of G3PDH protein was used as a loading control. For densitometric analysis of band intensity, a specific band on the enhanced chemiluminescence (ECL)-developed film was subjected to densitometric analysis (Adobe Photoshop). The densitometric readings were pooled and averaged from three independent experiments as described by us [35]. The background of densitometric reading on the ECL-developed film was subtracted.

### Statistical Analysis

All data are summarized as mean ± S.E. For the statistical analysis, Sigma Plot (SPSS, USA) was used for this study. A Student t-test was used to compare two experimental groups. A p value of <0.05 was considered statistically significant.

## Results

### Cigarette smoke exposure induces TTP downregulation in alveolar macrophage and epithelial cells

**Fig 1:**
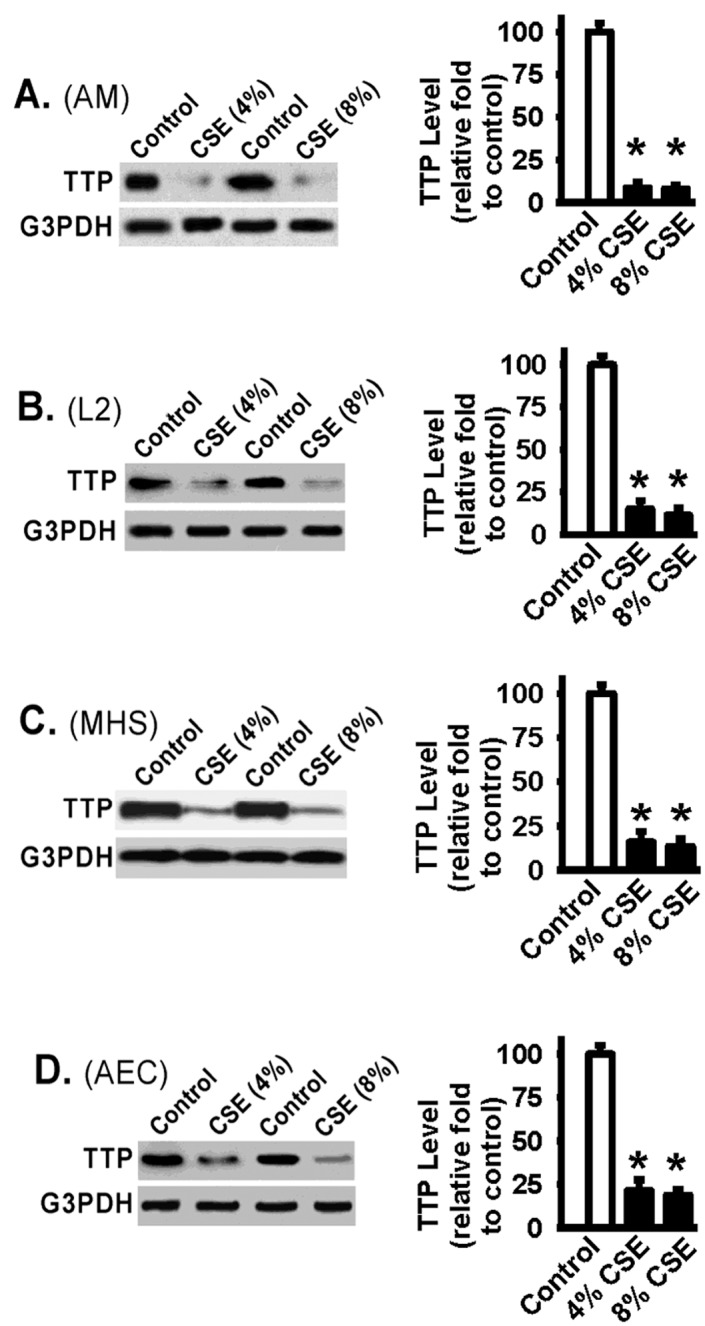
Cigarette smoke extract (CSE) decreases TTP in murine alveolar macrophages and epithelial cells. *Panel A*: Primary alveolar macrophages (AM) were incubated with CSE (4% and 8%, volume/volume in culture medium), or CSE control cell culture medium (Control: cigarettes were not burning), for 16 hours, then detergent lysed. Equivalent amounts of whole cell lysates were Western Blotted with the indicated antibodies. Glyceraldehyde 3-phosphate dehydrogenase (G3PDH) was used as a loading control for Western blot. *Panel B*: Rat alveolar epithelia cells (L2) were treated similarly as described in Panel A. Western Blots of whole cell lysates were performed and probed with indicated antibodies. *Panel C*: Murine alveolar macrophages (MHS) were treated similarly as described in Panel A. Western Blots of whole cell lysates were performed and probed with indicated antibodies. *Panel D*: Primary murine alveolar epithelial cells were treated similarly as described in Panel A. Western Blots of whole cell lysates were performed and probed with indicated antibodies. Representative images were shown. Densitometry analysis and data were pooled from at least three individual experiments for each group. * represents p < 0.01.

To understand the impact of cigarette smoke (CS) exposure on TTP function, we first examined TTP expression in alveolar macrophage and alveolar epithelial cells treated with CS extract (CSE). Primary alveolar macrophages and lung epithelial cells were obtained from C57Bl/6 mice. The rat alveolar epithelial cells (L2) and the mouse alveolar macrophages (MHS) were used for optimization of the test conditions and the experiments were repeated in primary alveolar macrophages and epithelial cells. Primary alveolar macrophages were treated with CSE (4% or 8% volume/volume) for 16 hours. Control primary alveolar macrophages were treated similarly with CSE control medium (cigarettes were not lit and not burnt). Western blot analysis of whole cell lysates showed that TTP level was significantly reduced in primary alveolar macrophages treated with CSE when compared to alveolar macrophages treated with CSE control medium (Fig 1A), supporting that CSE dampens the TTP in alveolar macrophages. Decreased TTP was also noted in alveolar epithelial cells (L2 cells, Fig 1B, Western blot analysis and densitometry), mouse alveolar macrophage cell line (MHS, Fig 1C, Western blot analysis and densitometry), and primary mouse alveolar epithelial cells (AEC, Fig 1D, Western blot analysis and densitometry). The results demonstrate that TTP is significantly down-regulated in response to CSE treatment in lung macrophage and epithelial cells.

### Cigarette smoke exposure increases the stability (half-life) of tumor necrosis factor alpha (TNF-α) mRNA in alveolar macrophages and alveolar epithelial cells

**Fig 2:**
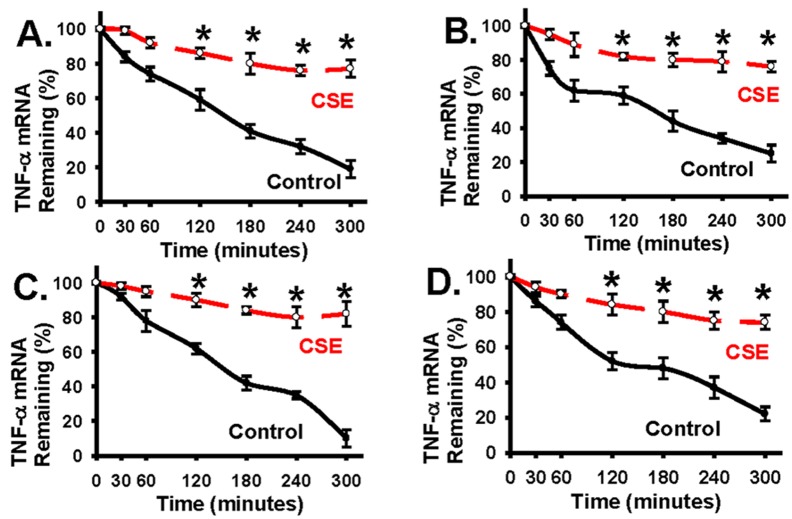
CSE treatment increases the stability and half-life of TNF-α mRNA. *Panel A*: Primary mouse alveolar macrophage (AM) were first treated with actinomycin D (10 μg/ml) to inhibit new transcription and then treated with CSE (CSE, 8% volume/volume) or with CSE control medium (Control: cigarettes were not burning) for the indicated time range (minutes). Total RNA at each time point was extracted from cells to determine the amount of TNF-α mRNA at each indicated time point (for stability and half-life) by real-time quantitative RT-PCR. Red dotted line (at the top) denotes the results from AMs treated with CSE. Black line (at the bottom) denotes the results from AMs treated with CSE-Control. *Panel B*: TNF-α mRNA stability and half-life in rat alveolar epithelial cells (L2) were examined as in Panel A. *Panel C*: TNF-α mRNA stability and half-life in mouse alveolar macrophages (MHS) were examined as in Panel A. *Panel D*: TNF-α mRNA stability and half-life in primary mouse alveolar epithelial cells were examined as in Panel A. Data are presented as mean ± S.E. (n = 3 individual samples). * represents p < 0.01 for the CSE-treated time points when compared to Controls (the top CSE line versus the bottom Control line).

Fig 1 shows that cigarette smoke extract (CSE) decreases the TTP protein level in lung macrophages and epithelial cells. It has been shown that TTP plays an important role in regulation of TNF-α mRNA stability [25;40]. TNF-α is a potent pro-inflammation mediator and its expression is up-regulated in COPD lungs [15–17]. Cigarette smoke induced TNF-α expression has been associated to the development of COPD [15–17]. To understand the functional consequence of CSE-induced TTP down-regulation on TNF-α expression, the mRNA stability of TNF-α was examined in alveolar macrophages and alveolar epithelial cells treated with CSE. Primary mouse alveolar macrophages were first treated with actinomycin D (10 μg/ml) to inhibit new transcription, then challenged with CSE (8% volume/volume) or CSE control medium (cigarettes were not burning) for the indicated time range (minutes, Fig 2A). Total RNA at each indicted time point was extracted from alveolar macrophages in order to determine the TNF-α mRNA stability over the tested time range, as well as the TNF-α mRNA half-life, by real-time quantitative RT-PCR. TNF-α mRNA half-life is about 140 minutes in primary alveolar macrophages treated with control medium (Fig 2A, the Control line). CSE treatment significantly increased the TNF-α mRNA half-life when compared to controls (Fig 2A, the CSE line versus the Control line, more remaining mRNA or high mRNA level means more stable). TNF-α mRNA stability was progressively decreased in control alveolar macrophages (Fig 2A, the Control line); in contrast, TNF-α mRNA stability was significantly increased in CSE-treated alveolar macrophages (Fig 2A, the CSE line).

Consistently, CSE treatment had similar effects on TNF-α mRNA half-life and stability in rat alveolar epithelial cells (Fig 2B), mouse alveolar macrophages (Fig 2C), and primary mouse alveolar epithelial cells (Fig 2D). The data (Fig 2) demonstrate that TNF-α mRNA half-life and stability are greatly increased in CSE treated macrophage and epithelial cells, likely due to CSE-induced downregulation of TTP in these macrophage and epithelial cells as shown in Fig 1. As TTP plays an important role to destabilize TNF-α mRNA [25;40], TTP-mediated TNF-α mRNA degradation is expected as an important mechanism to limit excessive inflammation in response to cigarette smoke and to prevent secondary lung tissue injury due to excessive inflammation response. Our findings suggest that TTP down-regulation caused by cigarette smoke exposure could contribute to persistent TNF-α expression observed in COPD lungs [15–17].

### Forced exogenous TTP expression abrogates CSE-induced increase of TNF-α mRNA stability and half-life in alveolar macrophages and alveolar epithelial cells

**Fig 3:**
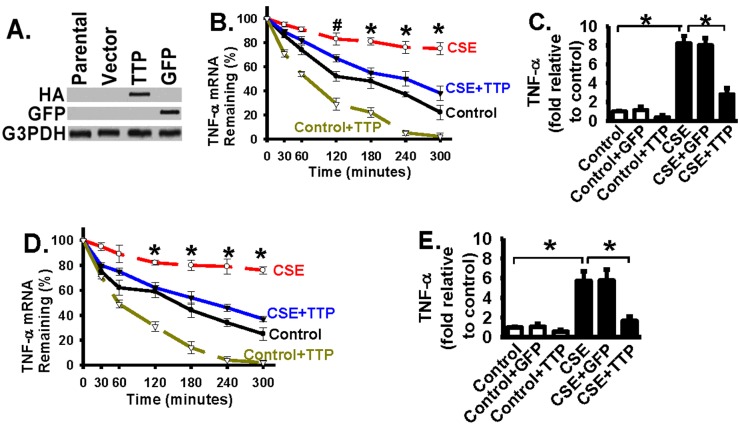
Forced exogenous TTP expression abrogates the CSE-induced increase of TNF-α mRNA half-life and production. *Panel A*: Forced exogenous TTP expression in primary mouse alveolar macrophages (AMs) was confirmed. Exogenous HA-tagged TTP expression was mediated by adenoviral vector in AMs. Whole cell lysates were western blotted with indicated antibodies and exogenous TTP expression was confirmed through the detection of the HA tag (top panel). Expression of green fluorescent protein (GFP) by adenoviral vector was used as a control. *Panel B*: TNF-α mRNA stability and half-life were examined in macrophages expressing TTP or GPF control in response to CSE challenge. AMs infected without or with adenoviral TTP vector (TTP) were treated with actinomycin D (10 μg/ml) to inhibit new transcription, and challenged with CSE (8% volume/volume) or with CSE control medium (Control: cigarettes were not burning) for the indicated time range (minutes). Total RNA was extracted from cells to determine the amount of TNF-α mRNA at each indicated time point (for stability and half-life) by real-time quantitative RT-PCR as in Fig 2. # represents p < 0.05 and * represents p < 0.01 for the CSE-treated group (CSE line) when compared to CSE + TTP group (CSE+TTP line). GFP expressing macrophages (GFP) were used as a control. *Panel C*: Cell culture medium was collected from indicated AM groups at 12 hours after CSE or CSE-Control treatment as in Panel B. GFP adenoviral vector served as a control for TTP adenoviral vector. The TNF-α protein level was measured by using the enzyme-linked immunosorbent assay (ELISA) kit for TNF-α according to the manufacturer’s instruction (R&D Systems). The basal TNF-a level in control-medium treated AMs (Control) is about 113 pg/ml. Data are represented as fold relative to the basal TNF-a level in control AMs. * represents p < 0.01. *Panel D*: TNF-α mRNA stability and half-life were examined in rat alveolar epithelial cells (L2) expressing TTP or GPF control in response to CSE challenge as in Fig 2. Cells were treated and TNF-α mRNA stability was examined similarly as in Panel B. * represents p < 0.01 for the CSE-treated group (CSE line) when compared to CSE + TTP group (CSE+TTP line). *Panel E*: The TNF-α protein levels in indicated epithelial cell groups were measured by ELISA as in Panel C. Data are represented as fold relative to control. * represents p < 0.01. GFP expressing vectors (GFP) were used as a control for TTP adenoviral vectors. All data are represented as mean ± S.E. (n = 3–4 individual samples).

The above data demonstrate that CSE induces TTP down-regulation (Fig 1), and CSE treatment results in increased stability and half-life of TNF-α mRNA in macrophages and epithelial cells (Fig 2). Therefore, we hypothesized that CSE-induced TTP down-regulation leads to the increased TNF-α mRNA stability and half-life in alveolar macrophages and alveolar epithelial cells. To test the hypothesis, the effects of gain of TTP function were examined. To achieve this, adenoviral vectors containing TTP cDNA with a hemagglutinin (HA) tag were generated in order to express exogenous TTP in alveolar macrophages and alveolar epithelial cells. Forced exogenous TTP expression was confirmed through detection of the HA tag in alveolar macrophages (Fig 3A, top panel) and in rat alveolar epithelial cells (L2) (data not shown). Adenoviral vectors expressing the green fluorescent protein (GFP) were used as a control for the adenoviral vector effect as previously described [41]. GFP expression mediated by adenoviral vectors was confirmed in alveolar macrophages (Fig 3A, top panel) and in rat alveolar epithelial cells (L2) (data not shown).

Forced expression of TTP (mediated by adenoviral vector) significantly reduced TNF-α stability and half-life in alveolar macrophages (Fig 3B, the CSE line vs. the CSE+TTP line). GFP expression (mediated by adenoviral vector) had no effect on TNF-α stability and half-life in alveolar macrophages (data not shown). Consequently forced expression of TTP significantly reduced the production of TNF-α protein in CSE-treated alveolar macrophages when compared to macrophages either treated with only CSE or CSE plus GFP expression (by adenoviral vector) (Fig 3C, bar 6 vs. bars 4 and 5). The basal TNF-a level in control-medium treated AMs (Control) was about 113 pg/ml. GFP adenoviral vector served as a control for TTP adenoviral vector. GFP expression had no effect on TNF-α levels (Fig 3C), indicating that the effect of exogenous TTP expression was specific. The TNF-α levels were measured by using the enzyme-linked immunosorbent assays (ELISA). These results demonstrate that forced exogenous TTP expression leads to the decreased mRNA stability and results in decreased protein expression of TNF-α in alveolar macrophages, supporting the important role of TTP in regulation of TNF-α expression in response to CSE exposure.

Similarly, forced expression of TTP significantly reduced TNF-α stability and half-life in rat alveolar epithelial cells (L2) (Fig 3D, the CSE line vs. the CSE+TTP line). Consequently, forced expression of TTP significantly reduced the TNF-α protein level in CSE-treated L2 cells when compared to that in L2 cells treated with only CSE or CSE plus GFP expression (by adenoviral vectors) (Fig 3E, bar 6 vs. bars 4 and 5).

CSE treatment induced TTP down-regulation (Fig 1) and increased the stability and half-life of TNF-α mRNA in macrophages and epithelial cells (Fig 2), leading to increased TNF-α protein expression (Fig 3C and 3E, bar 4 vs. bar 1). Forced exogenous TTP expression abrogated CSE-induced increase of TNF-α mRNA stability and subsequently significantly reduced TNF-α mRNA protein production (Fig 3). Taken together, these data support that TTP plays a critical role in regulation of cigarette smoke induced TNF-α expression at the mRNA level in lung macrophages and epithelial cells.

### TTP negatively regulates CSE-induced TNF-α expression at the mRNA level by directly targeting the 3’-AU rich region (ARE) of TNF-α mRNA and degrading TNF-α mRNA

**Fig 4:**
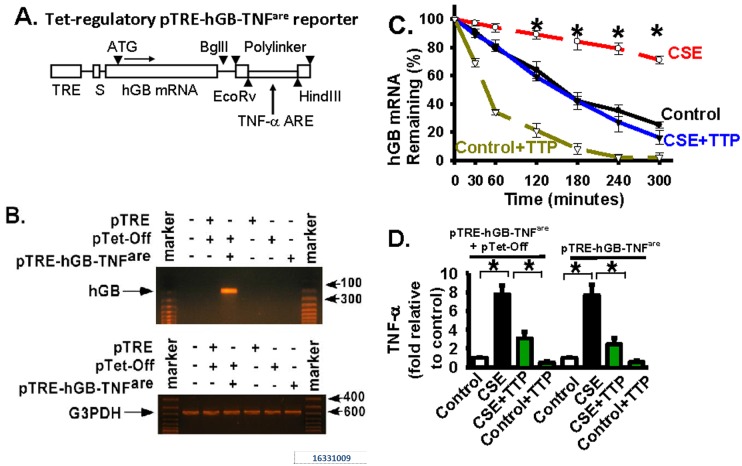
TTP negatively regulates CSE-induced TNF-α expression through directly binding the 3’-AU rich region (ARE) and degrading TNF-α mRNA. *Panel A*: The human TNF-α mRNA 3’-AU-rich element (ARE) (nucleotides 1221–1310, accession # M10988) was cloned into a tetracycline (Tet) regulated reporter plasmid between the EcoRv and HindII sites located at the end of polylinker (pTRE-hGB-TNF^are^ reporter). This reporter construct contains the tetracycline-response element (TRE), the puromycin selection marker (S), the human β-globin mRNA (hGB mRNA, as the reporter), and the TNF-α mRNA 3’-ARE. The stability of the TNF-α 3’-ARE can be examined through the hGB mRNA expression. *Panel B*: Primary alveolar macrophages (AMs) were infected with pTRE empty vector (pTRE), pTRE-hGB-TNF^are^ reporter vector, or/and pTet-Off vector as indicated. AMs were grown in tetracycline-free medium and the hGB mRNA expression was examined by PCR. Glyceraldehyde 3-phosphate dehydrogenase (G3PDH) mRNA expression served as a control for hGB mRNA expression. Co-infection of pTet-Off regulator plasmid and pTRE-hGB-TNF^are^ reporter induced the hGB expression. In this Tet-Off system, gene expression was turned on when tetracycline was removed from the culture medium. *Panel C*: The stability of the TNF-α 3’-ARE has been examined through the stability and expression of the hGB mRNA. The above AMs with both pTRE-hGB-TNF^are^ reporter and pTet-Off plasmids were grown in tetracycline-free medium. Then, AMs were infected with adenoviral TTP vector (TTP) to overexpress exogenous TTP, then treated with actinomycin D (10 μg/ml) to inhibit new transcription, cultured with CSE (8% volume/volume) or with CSE control medium (Control: cigarettes were not burning). Total RNA was extracted from cells at the indicated time points (minutes) to determine the amount of hGB mRNA by real-time quantitative RT-PCR as in Fig 2 (also to examine the stability and half-life). * represents p < 0.01 for the CSE-treated group (CSE line) when compared to CSE + TTP group (CSE+TTP line). *Panel D*: Cell culture medium was collected from the indicated AM groups at 12 hours after CSE or CSE control (Control) treatment as in Fig 3. The TNF-α protein level was measured by using the ELISA kit. Data were represented as fold relative to the basal TNF-a level in control AMs. * represents p < 0.01. All data are presented as mean ± S.E. (n = 3–4 individual samples).

The above data clearly indicate that TTP plays a critical role in regulation of cigarette smoke induced TNF-α production at the mRNA level in lung macrophages and epithelial cells. To understand the molecular mechanism involved, we have cloned the human TNF-α mRNA 3’-AU rich region (ARE) (nucleotides 1221–1310) into a tetracycline (Tet) regulated reporter vector (the pTRE-hGB-TNF^are^ reporter, Fig 4A). This reporter construct contains the tetracycline-response element (TRE), the human β-globin (hGB) mRNA (as the reporter), and the TNF-α mRNA 3’-ARE; therefore, whether TTP regulates TNF-α mRNA through the 3’-ARE can be confirmed through the expression and stability of the hGB mRNA. Primary mouse alveolar macrophages were infected with the pTRE-hGB-TNF^are^ reporter and pTet-Off regulator vectors; the human β-globin (hGB) reporter gene was expressed when cells were cultured in medium without tetracycline (the Tet-Off system). The hGB expression was confirmed by detection of hGB mRNA in primary mouse alveolar macrophages (Fig 4B, top panel, lane 4). The pTRE empty vector (pTRE) was used as a vector control for the pTRE-hGB-TNF^are^ reporter vector. hGB mRNA expression was not detected in cells infected with pTRE empty vector, pTet-Off, or pTRE-hGB-TNF^are^ reporter vector alone (Fig 4B, top panel), while each of the tested conditions showed the mRNA expression of glyceraldehyde 3-phosphate dehydrogenase (G3PDH) (Fig 4B, bottom panel). G3PDG expression served as a control in all conditions (Fig 4B). The results demonstrate that hGB reporter expression has been achieved and it is specific.

CSE treatment dramatically stabilized the hGB mRNA when compared to CSE control treatment in macrophages (Fig 4C, the CSE line vs. the Control line), indicating that the stability of the TNF-α mRNA 3’-ARE region was greatly increased in response to CSE treatment. CSE treatment significantly reduced TTP (Fig 1A) and increased TNF-α mRNA half-life and protein expression (Fig 2A) in macrophages. Data in Fig 4C demonstrate that TTP negatively regulates TNF-α expression at the mRNA level by directly binding to the 3’-AU rich region (ARE) of TNF-α mRNA and degrading TNF-α mRNA; therefore, TTP down-regulation induced by CSE leads to increased TNF-α mRNA stability and expression (evidenced by the hGB reporter expression).

To examine the effect of restored (forced exogenous) TTP expression on CSE-increased hGB mRNA stability, alveolar macrophages were infected with adenoviral TTP vectors and then treated with or without CSE. Forced exogenous expression of TTP significantly reduced hGB stability and half-life (Fig 4C, the CSE line vs. the CSE+TTP line). Significantly decreased hGB mRNA expression indicates that TTP negatively regulates the TNF-α mRNA stability and expression by directly targeting the 3’-ARE region of TNF-α mRNA in alveolar macrophages. Forced expression of TTP also significantly reduced hGB expression in control macrophages (treated with CSE-control medium) (Fig 4C, the Control+TTP line). Meanwhile, the production of TNF-α protein was confirmed in these macrophages, supporting that loss of TTP (induced by CSE) increased TNF-α expression while gain of TTP (by adenoviral vector) decreased TNF-α expression (Fig 4D).

**Fig 5:**
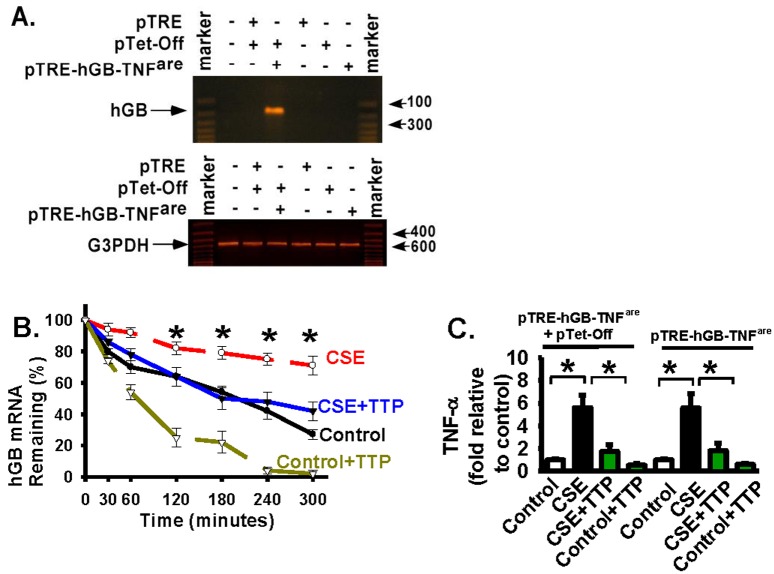
TTP destabilizes the 3’-AU-rich element (ARE) and degrades TNF-α mRNA in alveolar epithelial cells. *Panel A*: Rat alveolar epithelial cells (L2) were infected with pTRE empty vector, pTRE-hGB-TNF^are^ reporter vector, or/and pTet-Off vector as indicated. Cells were grown in tetracycline-free medium and the hGB mRNA expression was examined by PCR. G3PDH mRNA expression served as a control. *Panel B*: The above L2 cells with both pTRE-hGB-TNF^are^ reporter and pTet-Off plasmids were grown in tetracycline-free medium. Cells then were infected with adenoviral TTP vector (TTP), treated with actinomycin D (10 μg/ml) to inhibit new transcription, cultured with CSE (8% volume/volume) or with CSE control medium (Control: cigarettes were not burning). Total RNA was extracted from cells to determine the amount of hGB mRNA at the indicated time points (minutes) by real-time quantitative RT-PCR as in Fig 2 (to examine the stability and half-life). * represents p < 0.01 for the CSE-treated group (CSE line) when compared to CSE + TTP group (CSE+TTP line). *Panel C*: Cell culture medium was collected from indicated L2 cell groups at 12 hours after CSE or control treatment as in Fig 3. The TNF-α protein level was measured by using the ELISA kit. Data were represented as fold relative to the basal TNF-a level in control-treated cells. * represents p < 0.01. All data are presented as mean ± S.E. (n = 3–4 individual samples).

To examine whether similar mechanism exists in alveolar epithelial cells, rat alveolar epithelial cells (L2) were infected with the pTRE-hGB-TNF^are^ reporter and pTet-Off regulator vectors. The hGB reporter mRNA expression was confirmed in epithelial cells infected with both pTRE-hGB-TNF^are^ reporter and pTet-Off regulator vectors (Fig 5A). Similarly to macrophages, CSE treatment significantly stabilized the hGB mRNA when compared to CSE control (Fig 5B, the CSE line vs. the Control line). Forced exogenous expression of TTP (mediated by adenoviral vector) significantly reduced hGB mRNA stability and half-life (Fig 5B, the CSE line vs. the CSE+TTP line). The results demonstrate that TTP negatively regulates the TNF-α mRNA stability by directly targeting the 3’-ARE region of TNF-α mRNA. Consistently, CSE treatment increased the TNF-α protein level; forced TTP expression abrogated the CSE-increased production of TNF-α protein production in alveolar epithelia cells (Fig 5C). The findings are also consistent with the results shown in Fig 3, that CSE treatment increases the TNF-α production; in contrast, forced exogenous TTP expression abrogates the CSE-increased TNF-α expression in alveolar macrophages and epithelial cells (Fig 4D). Taken together, these data support that TTP negatively regulates TNF-α production at the post-transcriptional level through directly targeting TNF-α mRNA 3’-ARE region and degrading TNF-α mRNA.

## Discussion

Tobacco smoke has been firmly associated with the development of chronic obstructive pulmonary disease (COPD) [1–4]. COPD is a progressive lung disease and COPD patients have reduced lung function due to the abnormal permanent enlargement of airspaces and destruction of lung structure [1;4–6]. The pathological features of COPD include, but not limited to, substantial inflammation, fibrotic remodeling of the airways, and alveolar destruction [2–6]. The major contributing factor is thought to be persistent inflammation induced by cigarette smoke. Exposure to second hand tobacco smoke (environment tobacco smoke), the most important indoor air pollutant in an enclosed environment, also is firmly associated with inflammatory responses [42;43]. However, molecular mechanisms leading to the persistent inflammation in COPD lungs have not been completely defined. In order to develop novel therapeutic intervention to combat persistent inflammation induced by tobacco smoke exposure, it is important to understand the molecular mechanisms leading to persistent inflammation in lung induced by tobacco smoke exposure.

Tumor necrosis factor alpha (TNF-α) is one of the potent pro-inflammation mediators and its expression is up-regulated in COPD lungs [15–17]. Increased TNF-α expression has been firmly associated with persistent inflammatory responses in COPD lungs and evidences support that TNF-α plays an important role in cigarette smoke induced inflammation in COPD [8;15;16]. Regulation of mRNA stability is one important mechanism to control gene expression and the mechanisms involved are critical to limit excessive inflammation in response to tissue injury [18–20]; however, molecular mechanisms regulating TNF-α mRNA stability in response to cigarette smoke exposure are not well understood so far.

TTP is a widely distributed phospho-protein encoded by the immediate–early response gene [21–23]. TTP is best known as an mRNA-destabilizing protein [25–27]. The adenine- and uridine-rich element/region (named ARE) is a critical cis-acting regulatory motif located within the 3' untranslated regions (3'-UTR) of many mRNAs, including mRNAs of pro-inflammation cytokines. TTP can directly bind to the 3’-UTR ARE of targeted mRNAs, and promotes the degradation of the targeted mRNAs by recruiting the cellular mRNA degradation machinery to the targeted mRNAs [24–27;44]. Published studies show that TTP binds directly to the 3'-UTR ARE of the TNF-α mRNA, and destabilizes TNF-α mRNA (decreases the half-life) [19;21;27]. Therefore, TTP-mediated TNF-α mRNA destabilization pathway represents one key mechanism by which inflammation is limited and tightly controlled during tissue injury and repairing processes.

Although it is known that TTP negatively regulates TNF-α mRNA stability [27;36;45–48] and TNF-α is persistently upregulated and contributes to COPD pathogenesis [8;15;16], the effects of tobacco smoke exposure on TTP expression and TTP’s ability to regulate TNF-α expression in the context of cigarette smoke exposure are unknown. To understand the impact of cigarette smoke exposure on TTP function, we examined TTP expression in alveolar macrophage and alveolar epithelial cells treated in response to cigarette smoke extract. Cigarette smoke exposure induced significant TTP downregulation in alveolar macrophage and alveolar epithelial cells (Fig 1). Alveolar macrophage and alveolar epithelial cells are thought as two of major cell types contributing to the production of excessive pro-inflammatory mediators in response to cigarette smoke and during COPD development. Indeed, the downregulation of TTP expression induced by cigarette smoke exposure increased the TNF-α mRNA stability, extends TNF-α mRNA half-life), and significantly increases TNF-α protein levels in alveolar macrophages and alveolar epithelial cells (Figs 2 and 3). These data support a central role of TTP in modulation of TNF-α expression and that impaired TTP function caused by cigarette smoke exposure results in prolonged and persistent TNF-α expression.

Furthermore, ectopic expression of TTP significantly reduced TNF-α mRNA stability and half-life in alveolar macrophages and epithelial cells (Fig 3). The data clearly support the role of TTP in regulation of TNF-α at the post-transcriptional level and suggest that intact TTP expression and function is required to limit the extent of TNF-α expression induced by external stimulants. TTP is likely playing an important role or acts as one of the critical checkpoints to prevent prolonged and excessive expression of pro-inflammatory mediators in response to toxic stimulants such as cigarette smoke. This is important to limit tissue injury during tissue repairing process. TTP also plays an important role in regulation of other mediators which are important in inflammation and angiogenesis, such as cyclooxygenase (COX-2) [49;50], vascular endothelial growth factor (VEGF) [28], mouse chemokine KC (CXCL1) [51], granulocyte-macrophage colony-stimulating factor (GM-CSF) [52], and interleukin 4 (IL-4) [53]. The generation of TTP-deficient mice further supports the role of TTP in destabilization of TNF-α mRNA, and the role of TTP as an anti-inflammatory protein *in vivo* [27]. Mice deficient in TTP develop a severe syndrome characterized by growth retardation and cachexia, arthritis, dermatitis, and autoimmunity [27]. Treatment of TTP-deficient mice with neutralizing anti-TNF-α antibodies results in alleviating most of the pathological features in TTP-deficient mice [27], demonstrating the involvement of TNF-α in the development of the pathological features in TTP-deficient mice. The effect of loss of TTP function on inflammation in response to cigarette smoke exposure *in vivo* has not been examined in detail and is a focus of the future work. Given the evidence suggesting that TTP is one major regulator of the expression of TNF-α and other mediators [28;49–53] and that persistent inflammation is firmly associated with COPD development [4;7–10], TTP deficiency is likely to magnify the detrimental effect of cigarette smoke exposure, including second hand smoke, on lung tissue injury.

**Fig 6:**
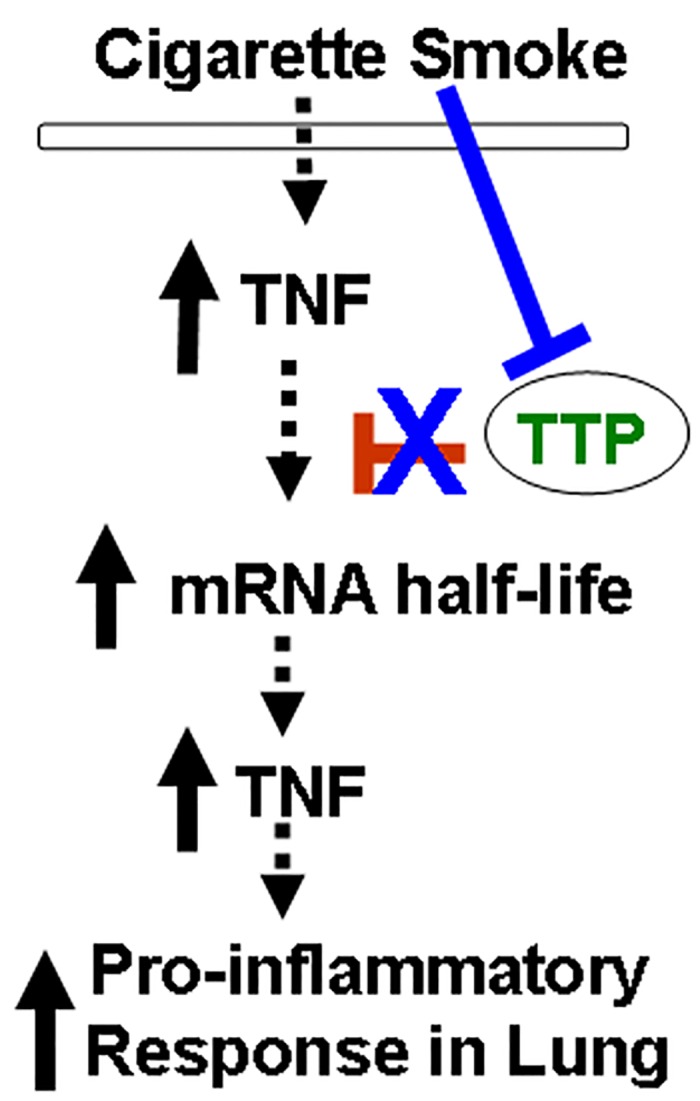
Proposed schematics of TTP-mediated mechanism of regulation of TNF-α expression and cigarette smoke exposure causes increased TNF-α expression by TTP downregulation. TTP negatively regulates TNF-α expression through a post-transcription mechanism by directly targeting the ARE regions of TNF-α mRNA and negatively regulates TNF-α expression at the post-transcriptional level. TTP functions to reduce TNF-α mRNA stability and half-life; thereby, through post-transcription regulation mechanism, TTP limits the extent of TNF-α expression and preventing tissue injury due to excessive and prolong inflammatory responses. Cigarette smoke exposure reduces TTP expression and that leads to significantly increased stability of TNF-α mRNA and persistent TNF-α expression, which has been firmly associated with tissue injury and COPD pathogenesis.

Regulation of the expression of pro-inflammation mediators is complicated and it is likely mediated by multiple pathways, either directly or indirectly [2;54–58]. It has been demonstrated that TTP regulates TNF-α expression through mRNA destabilization [23;27;59;60]. Since other pathways also induces TNF-α expression [2;54–58;61], whether TTP-mediated TNF-α expression is the major regulatory pathway contributing to TNF-α expression in response to cigarette smoke exposure, and whether it is directly through the mRNA ARE domain, have to be tested. By using the tetracycline (Tet) regulated reporter vector (the pTRE-hGB-TNF^are^ reporter), this study demonstrates that TTP negatively regulates TNF-α expression at the mRNA level by directly targeting the 3’-AU rich region (ARE) of TNF-α mRNA and degrading TNF-α mRNA (Fig 4). This mechanism is consistent with previously published mechanism regarding to the role of TTP in regulation of TNF-α mRNA stability and expression. Importantly, this study shows that TTP is critical in defending and preventing secondary tissue injury due to excessive and prolonged inflammatory storm in certain disease development, such as COPD. In fact, there is clear evidence to support the pathobiologic role of TNF-α in COPD, and asthma, mainly in severe refractory asthma. TNF-α inhibitors (such as infliximab, golimumab and etanercept) are now considered as the potential new medications in COPD and asthma management [61]. This study suggests a novel role and the mechanism of TTP in cigarette smoke induced inflammation in alveolar macrophages and epithelial cells (Fig 6). As persistent inflammation and increased TNF-α expression have been contributing to the development of COPD [4;7–11], understanding the molecular mechanisms of TTP downregulation in response to cigarette smoke exposure will definitely benefit the field and could provide new insights regarding therapeutic intervention to reduce inflammation cell recruitment, epithelial cell death, and abnormal enlargement of airspace in COPD patients.

In summary, we demonstrate in this study that cigarette smoke exposure induces TTP down-regulation in alveolar macrophage and alveolar epithelial cells. Reduced TTP expression leads to significantly increased stability of TNF-α mRNA and TNF-α expression in macrophages and epithelial cells exposed to cigarette smoke extract. Forced TTP expression abrogates the increased stability of TNF-α mRNA resulting in significantly reduced TNF-α expression in these cells. Importantly, the data demonstrate that TTP limits excessive TNF-α expression through a post-transcription mechanism by directly targeting the ARE regions of TNF-α mRNA and negatively regulates TNF-α expression at the post-transcriptional level. Taken together, the data demonstrate that TTP is a novel post-transcriptional regulator and limits the TNF-α expression and pro-inflammatory response induced by cigarette smoke. Cigarette smoke exposure impairs TTP function resulting in prolonged TNF-α mRNA stability and that subsequently leads to persistent TNF-α expression, which has been firmly associated with tissue injury and COPD pathogenesis.
